# Determinants of peripapillary retinal nerve fiber layer’s grayscale value in normal eyes by spectral domain optical coherence tomography

**DOI:** 10.1038/s41598-021-88604-x

**Published:** 2021-05-05

**Authors:** Xiaolin Xie, Binyao Chen, Jianling Yang, Chukai Huang, Kunliang Qiu, Ce Zheng, Mingzhi Zhang

**Affiliations:** 1 Joint Shantou International Eye Center of Shantou University and the Chinese University of Hong Kong, Shantou University Medical College, Shantou, 515000 Guangdong China; 2 Department of Ophthalmology, Xinhua Hospital, Affiliated to Shanghai Jiaotong University School of Medicine, Shanghai, China

**Keywords:** Diagnostic markers, Computational biophysics

## Abstract

To determine and evaluate the distribution, variation, and determinants of peripapillary retinal nerve fiber layer (pRNFL) grayscale value with spectral-domain optical coherence tomography (SD-OCT) in normal eyes. In this cross-sectional study, three hundred ninety-seven normal eyes from 397 healthy Chinese adults aged 18–80 were consecutively recruited from a tertiary eye care center. An SD-OCT instrument took pRNFL imaging. We used a customized software to measure pRNFL parameters, including thickness and grayscale value. Univariable and multiple linear regression analyses were performed to examine the relationship between pRNFL grayscale value with ocular (e.g., axial length [A.L.], spherical equivalent [S.E.], intraocular pressure [IOP]), and systemic (e.g., age, sex) factors. A total of 397 eyes from 397 healthy subjects were included in the final analysis with mean (± SD) age 44.63 ± 16.43 years (range 18–80 years) and 196 (49.4%) males. The mean average of pRNFL grayscale value and thickness 164.82 ± 5.69 and 106.68 ± 8.89 μm, respectively. pRNFL grayscale value in nasal sectors (163.26 ± 9.31) was significantly lower comparing those in all other five sectors (all with *p* < 0.001)]. In multivariable analysis, average pRNFL grayscale value was independently correlated to older age (β = − 0.053, *p* = 0.002), longer axial length (β = − 0.664, *p* = 0.003), lower RPE grayscale value (β = 0.372, *p* < 0.001) and lower ImageQ (β = 0.658, *p* < 0.001). In this study, we provided normative SD-OCT data on the pRNFL grayscale value profile in nonglaucomatous eyes. Lower average pRNFL grayscale value was independently correlated to older age, longer axial length, lower RPE grayscale value, and lower ImageQ. These determinants should be considered when interpreting pRNFL grayscale value in glaucoma assessment.

## Introduction

Several studies have suggested that structural change of the peripapillary retinal nerve fiber layer (pRNFL) is an early sign of glaucoma, which is a leading cause of irreversible blindness worldwide^1^. Since the advance of Spectral-Domain Optical coherence tomography (SD-OCT), it now possible to assess pRNFL morphological features, such as thickness or volume^2^. Kuang et al. reported that SD-OCT's pRNFL thickness measurements might be able to detect glaucomatous damage 5–6 years before the appearance of the earliest visual field defects. Mwanza et al. demonstrated that pRNFL thickness parameters, including thickness in different quadrants, have excellent ability to discriminate between normal eyes and eyes with even mild glaucoma^3^.

Although morphological features of pRNFL show valuable clinical application in glaucoma detection, some authors also mentioned that morphological features do not fully exploit the potential of this SD-OCT modality^4^. Recently, we proposed a deep learning model for automated detection of glaucoma and achieved higher sensitivity and specificity compared to standard pRNFL morphological features^5^. Deep learning can learn representations of medical images with multiple levels of abstraction^6^. This result suggested that other SD-OCT features, besides pRNFL thickness, may also play a role as a sign of glaucomatous damage. Schoot et al. confirmed the diminished RNFL attenuation coefficient in glaucomatous eyes compared to healthy eyes in SD-OCT images^7^. Vermeer et al. reported a significant difference of pRNFL optical intensity in SD-OCT images between normal and glaucomatous eyes^4^.

Previous studies have shown that pRNFL thickness was influenced by determinants such as age, sex, ethnicity, axial length, and image quality on normal subjects using SD-OCT^8–10^. Nonetheless, it remains unknown whether these determinants also have any significant influence on pRNFL grayscale value measurements. It is critical to establish a normative database, as the application of newly developed features depends on understanding normal conditions. ﻿Therefore, our study aimed to examine the influences of demographic, ocular, and systemic factors on pRNFL grayscale value measurements using SD-OCT in nonglaucomatous Chinese adult subjects.

## Methods

In this cross-sectional study, Chinese subjects above 18 years were consecutively recruited from the Joint Shantou International Eye Center of Shantou University and the Chinese University of Hong Kong (JSIEC), a tertiary eye care center in south China, between September 2013 and March 2015. Ethics committee approval was obtained from the Institutional Review Board (identifier, EC 20130927(2)-P07) of JSIEC. This study was conducted according to the tenets of the Declaration of Helsinki, and written informed consent was obtained from each participant.

### Study participants

Normal participants included hospital staff or subjects who conducted a routine eye examination. After asking their medical and ophthalmic history, all participants underwent a standardized ophthalmic examination, which included: (1) slit-lamp biomicroscope (model BQ-900; Haag-Streit, Switzerland); (2) best-corrected V.A. (BCVA) with Early Treatment of Diabetic Retinopathy Study (ETDRS) chart; (3) intraocular pressure (IOP) measurement by Goldmann applanation tonometry; (4) fundus examination with a 78-D lens (Volk Optical, USA); (5) axial length measured with the IOL Master 500 (software number 7.1.2.0042, Carl Zeiss Meditec); (6) refractive error assessed from an autorefractor (Canon RK-F1, Japan) followed by subjective refraction; (7) V.F. testing with﻿ Swedish interactive threshold algorithm fast 24-2 (Humphrey Field Analyzer II-750i, Carl Zeiss Meditec). A visual field (V.F.) was defined as reliable when fixation losses were less than 20%, and false-positive and false-negative rates were less than 33%. Normal V.F. was defined as mean deviation (M.D.) and pattern standard deviation (PSD) within 95% confidence limits, and a glaucoma hemifield test (GHT) result within normal limits. Exclusion criteria were: BCVA > 0.3 [logarithmic minimal-angle resolution (LogMAR)], spherical equivalent (spherical error + ½ of cylindrical error) less than -6.0 D or more than 6 D, previous retinal or refractive surgery, neurologic diseases, or clinical features compatible with a diagnosis of a glaucoma suspect or glaucomatous visual field defect. Glaucomatous V.F. defects were defined as those with a cluster of three points with probabilities of < 5% on the pattern deviation map in at least one hemifield, including at least 1 point with a probability of < 1%; or a cluster of two points with a probability of < 1%, and a GHT result outside 99% of age-specific normal limits or a PSD outside 95% of normal limits.

### SD-OCT imaging and imaging analysis

Topcon 3D OCT-2000 (Topcon, Tokyo, Japan, software version: 8.11.003.04) is a commercially available SD-OCT device. It has an acquisition rate of 20,000 A-scans per second. The transverse and axial resolutions were 20 and 5 μm, respectively. All subjects received scans by experienced operators (X.L, B.C, and J.Y.) without pupil dilatation. Each eye was imaged using the optic disc 3D protocol (1024 points of resolution on a 3.46 mm circle diameter). pRNFL images were exported and saved in jpg format for quantitative analysis. Only images with a quality factor > 45 were used for studies.

**Figure 1:**
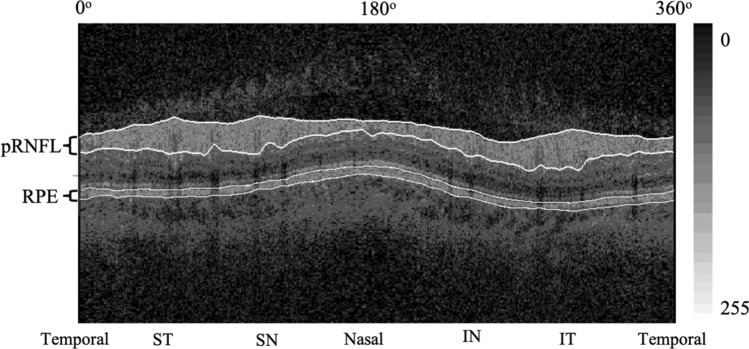
SD-OCT images of the pRNFL and RPE segmentation with grayscale value measurement.

We developed a customized software (Anterior Segment Analysis Program (ASAP)) to measure pRNFL parameters, including thickness and grayscale value automatically^5^. ASAP was coded as a plug-in software under ImageJ (version 1.38x), a public domain Java program (available at http://rsb.info.nih.gov/ij, National Institutes of Health, Bethesda, MD, USA). The software first automatically detected the pRNFL and RPE's boundary. One ophthalmologist (XL.X with five years of experiments) inspected every OCT image and excluded images with boundary misidentification. The detail of grayscale value had been reported by other research groups and us. In this study, the grayscale value was defined as the gray value range from 0 (pure black) to 255 (pure white). The ASAP software then automatically calculated the pRNFL parameters on average and six different sectors after automatically delineating retinal structure boundary, like pRNFL and RPE. pRNFL parameters evaluated in this study were the average thickness and grayscale value in 360°, with 315°–45° position designated temporal, 270°–315° position inferior temporal, 225°–270° position inferior nasal, 135°–225° position nasal, 90°–135° position superior nasal and 45°–90° position superior temporal (Fig. 1). A subset of 20 images was randomly selected for assessing the inter-observer reproducibility. 2 examiners (X.L and C.Z) independently measured pRNFL parameters using ASAP. The intra-class correlation coefficient ranged from 0.74 to 0.95 for all the pRNFL measurements in the current study.

### Statistical analysis

Continuous variables, including demographic characteristics and ocular features, were described as the mean, standard deviation, and range. Bonferroni corrections were applied to multiple comparisons. Univariable and multivariable regression analyses were performed to determine demographic characteristics and ocular features (independent variables) associated with pRNFL grayscale value measurements (dependent variables). The significant variables (*p* < 0.05) in univariable analysis were included in multivariable analysis. All statistical analysis was performed using commercial analytic software (SPSS version 17.0; SPSS, Inc., Chicago, IL).

### Ethical approval

All procedures performed in this study were in accordance with the ethical standards of the institutional research committee and with the 1964 Helsinki Declaration and its later amendments.

## Results

Four hundred fifteen Chinese adults were recruited and underwent pRNFL imaging with SD-OCT. Among those, 18 (4.3%) were excluded because of boundary misidentification (n = 7, 1.7%) and motion artifact (n = 11, 2.6%). A total of 397 eyes from 397 healthy subjects were included in the final analysis: 196 (49.4%) males vs. 201 (50.6%) females. Table 1 summarizes the demographic characteristics and ocular features. The mean age was 44.63 years (range 18–80 years). The mean spherical equivalent was -0.78 ± 1.85 diopters (D), and the mean axial length was 23.73 ± 1.13 mm.

**Table 1:** Demographic characteristics and ocular features of the study participants (n = 397).

Variables	All (n = 397)	
	Mean ± SD	Range
Age (y)	44.63 ± 16.43	18–80
Sex (% male)	49.4	
Spherical equivalent (D)	− 0.78 ± 1.85	− 6.00 to + 3.50
Best corrected visual acuity (logMAR)	0.006 ± 0.075	− 0.2 to 0.3
Axial length (mm)	23.73 ± 1.13	20.7–26.0
IOP (mmHg)	14.43 ± 2.87	7.9–21
Visual field MD (dB)	− 1.01 ± 1.37	− 2.47 to 2.75
ImageQ	55.4 ± 4.35	45–65

**Figure 2:**
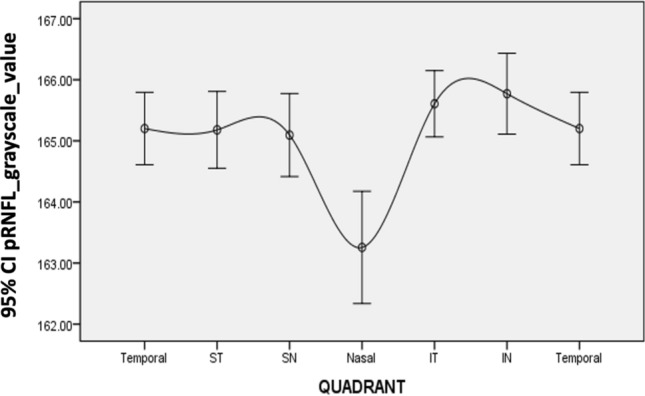
Sectoral distribution of pRNFL grayscale value of all study subjects.

**Figure 3:**
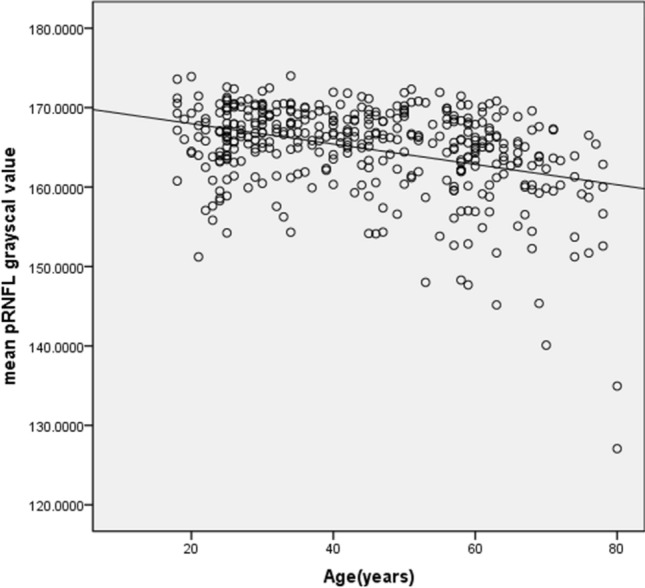
Scatterplot of mean pRNFL grayscale value against the age of study subjects (years).

**Figure 4:**
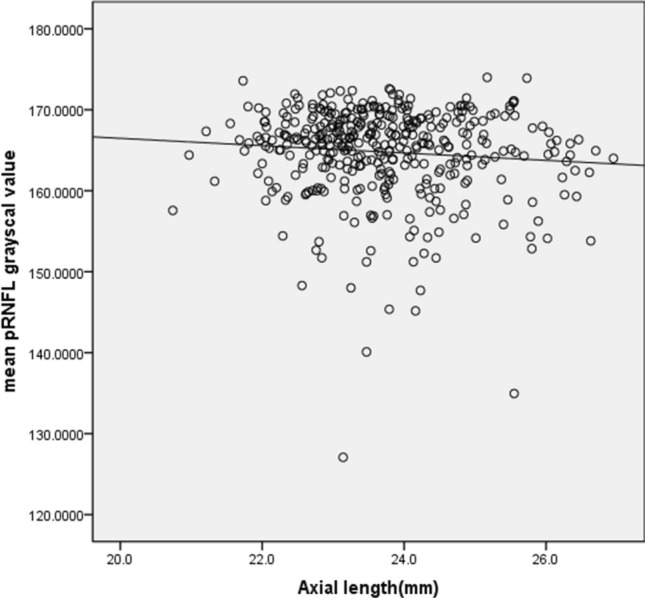
Scatterplot of mean pRNFL grayscale value against axial length.

**Figure 5:**
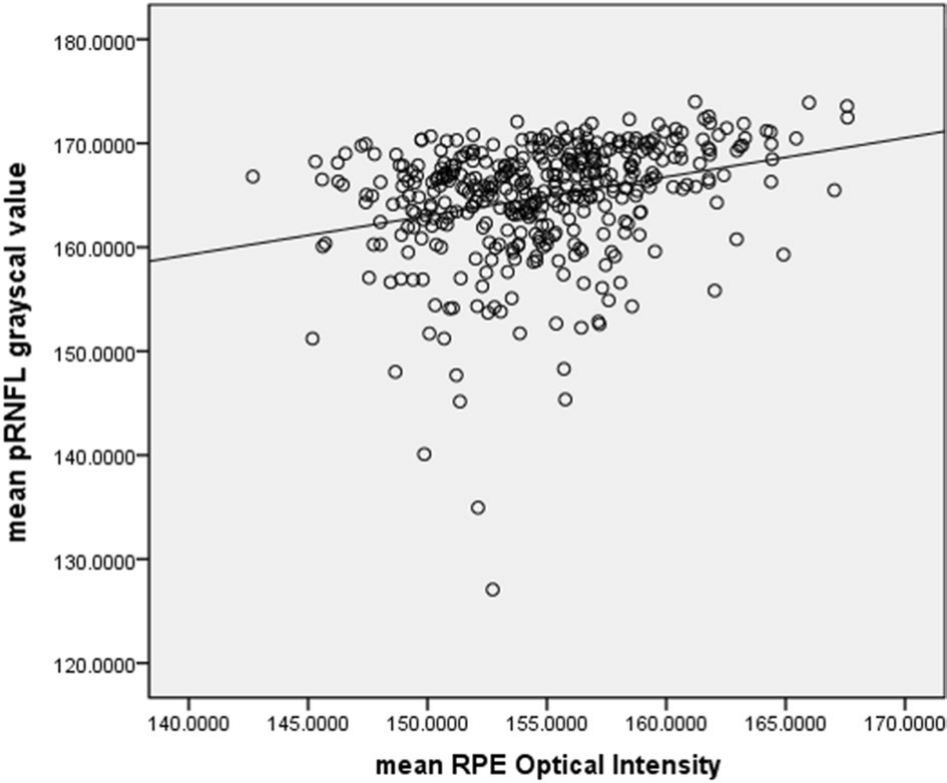
Scatterplot of mean pRNFL grayscale value against RPE grayscale value.

**Figure 6:**
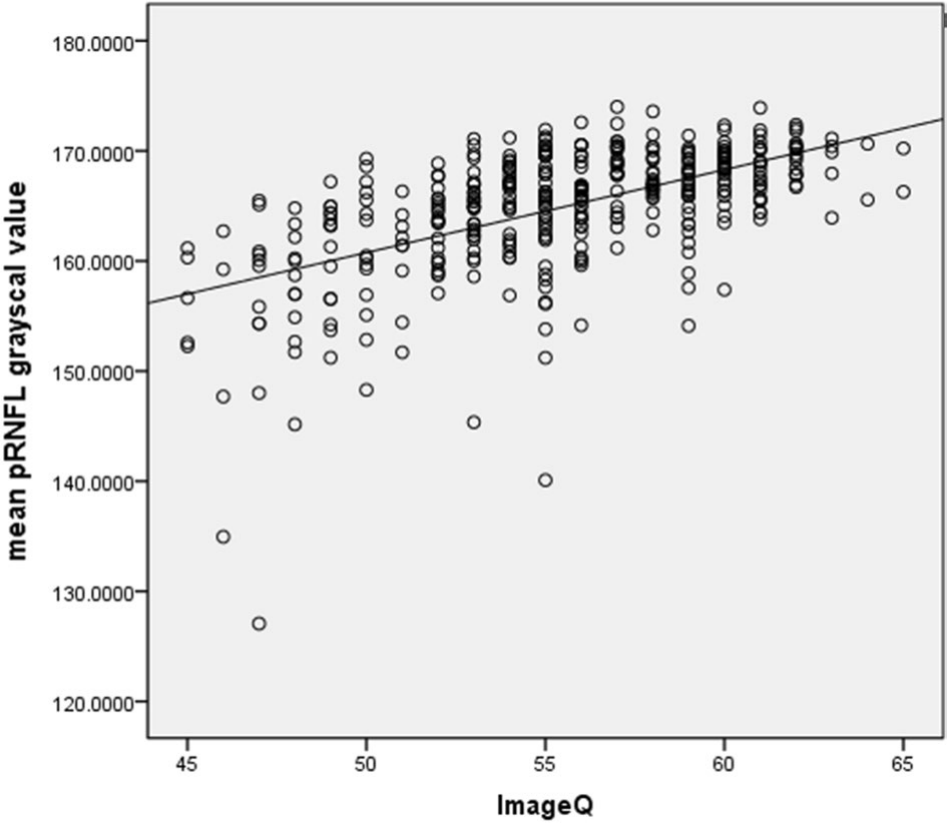
Scatterplot of mean pRNFL grayscale value against ImageQ.

The mean and SD of the pRNFL parameters of each age group are summarized in Table 2. The mean average of pRNFL grayscale value and thickness 164.82 ± 5.69 and 106.68 ± 8.89 μm, respectively. pRNFL grayscale value in nasal sectors (163.26 ± 9.31) was significantly lower comparing those in all other five sectors (all with *p* < 0.001, statistically significant after Bonferroni correction)] (Fig. 2). Table 3 shows the univariable analysis of the demographic characteristics and ocular features associated with pRNFL grayscale value and thickness. A lower average pRNFL grayscale value was significantly correlated with older age (β = − 0.370, *p* < 0.001) (Fig. 3), male (β = − 0.155, *p* = 0.002), better best corrected visual acuity ((β = − 0.294, *p* < 0.001), thinner pRNFL thickness (β = 0.257, *p* < 0.001), lower RPE grayscale value (β = 0.284, *p* < 0.001) (Fig. 4) and lower ImageQ (β = 0.575, *p* < 0.001) (Fig. 5). The significant parameters in univariable regression carried forward for multivariable analysis. As previous studies have shown that axial length correlated with pRNFL thickness^11^, we therefore added axial length in multivariable analysis as well. Table 4 shows multivariable analysis of pRNFL grayscale value after adjustment of associated factors. A lower average pRNFL grayscale value was independently correlated to older age (β = − 0.053, *p* = 0.002), l onger axial length (β = − 0.664, *p* = 0.003), lower RPE grayscale value (β = 0.372, *p* < 0.001) and lower ImageQ (β = 0.658, *p* < 0.001) (Fig. 6).

**Table 2:** Mean (± SD) of pRNFL parameters by age groups.

Age group (y)	Number	Mean	Temporal	Inferior-temporal	Inferior-nasal	Superior-temporal	Superior-nasal	Nasal
**(a) pRNFL grayscale value**								
18–29	98	166.18 ± 4.31	166.40 ± 4.41	166.78 ± 5.07	167.23 ± 5.78	166.77 ± 4.44	167.73 ± 4.42	164.04 ± 7.99
30–39	70	166.86 ± 3.75	166.49 ± 3.96	167.42 ± 4.23	167.98 ± 4.21	167.82 ± 3.64	167.64 ± 3.79	165.52 ± 7.42
40–49	68	165.71 ± 4.16	165.69 ± 5.02	166.42 ± 3.76	166.36 ± 5.35	166.43 ± 3.70	166.23 ± 4.35	164.43 ± 7.92
50–59	70	164.46 ± 5.89	165.10 ± 7.07	165.64 ± 4.44	165.23 ± 6.14	164.43 ± 6.03	163.48 ± 7.67	163.36 ± 10.07
60–69	66	163.01 ± 5.40	163.67 ± 6.66	163.83 ± 4.70	164.08 ± 5.79	163.15 ± 6.10	162.80 ± 6.02	161.42 ± 9.28
≥ 70	25	157.18 ± 9.99	159.91 ± 9.56	158.31 ± 10.40	158.28 ± 13.69	155.58 ± 12.78	155.09 ± 12.75	155.18 ± 14.65
Total	397	164.82 ± 5.69	165.20 ± 6.01	165.61 ± 5.51	165.77 ± 6.71	165.09 ± 6.37	165.09 ± 6.88	163.26 ± 9.31
**(b) pRNFL thickness**								
18–29	98	107.93 ± 8.16	92.83 ± 16.95	149.07 ± 16.33	119.04 ± 17.85	136.71 ± 14.19	123.53 ± 14.19	75.14 ± 15.88
30–39	70	108.09 ± 7.44	85.57 ± 10.90	146.89 ± 14.80	125.35 ± 19.34	135.68 ± 13.80	128.40 ± 17.53	78.36 ± 16.46
40–49	68	108.41 ± 7.84	83.93 ± 14.00	145.92 ± 13.64	126.38 ± 16.64	133.45 ± 14.62	126.77 ± 15.23	83.37 ± 14.35
50–59	70	107.53 ± 9.81	81.37 ± 10.72	144.14 ± 12.26	129.37 ± 20.11	132.06 ± 14.55	126.00 ± 21.08	82.81 ± 15.59
60–69	66	102.62 ± 9.03	78.94 ± 11.13	137.80 ± 13.73	121.59 ± 17.15	124.05 ± 15.96	118.58 ± 14.55	80.41 ± 12.88
≥ 70	25	101.52 ± 10.45	78.16 ± 8.00	132.13 ± 20.70	120.40 ± 22.05	125.17 ± 15.96	119.52 ± 23.85	79.00 ± 12.29
Total	397	106.68 ± 8.89	84.77 ± 13.97	144.34 ± 15.55	123.74 ± 18.78	132.32 ± 15.27	124.30 ± 18.09	79.59 ± 15.23

**Table 3:** Univariable analysis between demographic characteristics and ocular features with pRNFL grayscale value and thickness.

Variables, n = 397	Mean	Temporal	Inferior-temporal	Inferior-nasal	Superior-temporal	Superior-nasal	Nasal
**(a)**							
Age (y)	− 0.370 (*p* < 0.001)	− 0.276 (*p* < 0.001)	− 0.347 (*p* < 0.001)	− 0.313 (*p* < 0.001)	− 0.404 (*p* < 0.001)	− 0.449 (*p* < 0.001)	− 0.207 (*p* < 0.001)
Sex (% male)	− 0.155 (*p* = 0.002)	− 0.051 (*p* = 0.307)	− 0.132 (*p* = 0.008)	− 0.110 (*p* = 0.029)	− 0.187 (*p* < 0.001)	− 0.169 (*p* = 0.001)	− 0.152 (*p* = 0.002)
Spherical equivalent (D)	− 0.043 (*p* = 0.394)	− 0.005 (*p* = 0.919)	− 0.017 (*p* = 0.729)	− 0.015 (*p* = 0.771)	− 0.044 (*p* = 0.384)	− 0.084 (*p* = 0.093)	0.154 (*p* = 0.002)
Best corrected visual acuity (logMAR)	− 0.294 (*p* < 0.001)	− 0.224 (*p* = 0.001)	− 0.246 (*p* < 0.001)	− 0.224 (*p* < 0.001)	− 0.307 (*p* < 0.001)	− 0.310 (*p* < 0.001)	− 0.200 (*p* < 0.001)
Axial length (mm)	− 0.089 (*p* = 0.079)	0.014 (*p* = 0.777)	− 0.007 (*p* = 0.888)	− 0.070 (*p* = 0.166)	− 0.036 (*p* = 0.480)	0.004 (*p* = 0.933)	− 0.190 (*p* < 0.001)
IOP (mmHg)	− 0.002 (*p* = 0.974)	− 0.020 (*p* = 0.696)	− 0.017 (*p* = 0.736)	− 0.030 (*p* = 0.557)	− 0.019 (*p* = 0.709)	0.015 (*p* = 0.768)	− 0.012 (*p* = 0.805)
Visual field MD (dB)	0.083 (*p* = 0.100)	0.067 (*p* = 0.180)	0.080 (*p* = 0.112)	0.056 (*p* = 0.267)	0.066 (*p* = 0.192)	0.035 (*p* = 0.489)	0.079 (*p* = 0.114)
pRNFL thickness (um)	0.257 (*p* < 0.001)	− 0.041 (*p* = 0.420)	0.206 (*p* < 0.001)	0.230 (*p* < 0.001)	0.224 (*p* < 0.001)	0.297 (*p* < 0.001)	0.273 (*p* < 0.001)
RPE grayscale value	0.284 (*p* < 0.001)	0.284 (*p* < 0.001)	0.317 (*p* < 0.001)	0.226 (*p* < 0.001)	0.306 (*p* < 0.001)	0.291 (*p* < 0.001)	0.123 (*p* = 0.014)
ImageQ	0.575 (*p* < 0.001)	0.330 (*p* < 0.001)	0.471 (*p* < 0.001)	0.508 (*p* < 0.001)	0.491 (*p* < 0.001)	0.517 (*p* < 0.001)	0.511 (*p* < 0.001)
**(b)**							
Age (y)	− 0.260 (*p* < 0.001)	− 0.342 (*p* < 0.001)	− 0.312 (*p* < 0.001)	0.032 (*p* = 0.528)	− 0.294 (*p* < 0.001)	− 0.118 (*p* = 0.019)	0.120 (*p* = 0.017)
Sex (% male)	− 0.079 (*p* = 0.117)	− 0.142 (*p* = 0.005)	− 0.185 (*p* < 0.001)	− 0.070 (*p* = 0.165)	− 0.092 (*p* = 0.068)	− 0.108 (*p* = 0.031)	− 0.080 (*p* = 0.112)
Spherical equivalent (D)	− 0.089 (*p* = 0.075)	− 0.334 (*p* < 0.001)	− 0.120 (*p* = 0.017)	0.280 (*p* < 0.001)	− 0.067 (*p* = 0.186)	0.173 (*p* = 0.001)	0.323 (*p* < 0.001)
Best corrected visual acuity (logMAR)	− 0.199 (*p* < 0.001)	− 0.165 (*p* = 0.001)	− 0.193 (*p* < 0.001)	− 0.055 (*p* = 0.274)	− 0.158 (*p* = 0.002)	− 0.115 (*p* = 0.022)	− 0.041 (*p* = 0.419)
Axial length (mm)	− 0.158 (*p* = 0.002)	0.353 (*p* < 0.001)	0.063 (*p* = 0.214)	− 0.400 (*p* < 0.001)	0.059 (*p* = 0.242)	− 0.221 (*p* < 0.001)	− 0.357 (*p* < 0.001)
IOP (mmHg)	0.040 (*p* = 0.426)	0.066 (*p* = 0.189)	0.021 (*p* = 0.677)	− 0.012 (*p* = 0.809)	0.021 (*p* = 0.676)	− 0.039 (*p* = 0.445)	0.057 (*p* = 0.260)
Visual field MD (dB)	0.099 (*p* = 0.050)	− 0.010 (*p* = 0.844)	0.070 (*p* = 0.165)	0.044 (*p* = 0.377)	0.099 (*p* = 0.048)	0.082 (*p* = 0.102)	0.082 (*p* = 0.102)
pRNFL grayscale value	0.257 (*p* < 0.001)	− 0.095 (*p* = 0.058)	0.180 (*p* < 0.001)	0.251 (*p* < 0.001)	0.134 (*p* = 0.008)	0.304 (*p* < 0.001)	0.196 (*p* < 0.001)
ImageQ	0.412 (*p* < 0.001)	0.093 (*p* = 0.065)	0.223 (*p* < 0.001)	0.296 (*p* < 0.001)	0.4297 (*p* < 0.001)	0.311 (*p* < 0.001)	0.254 (*p* < 0.001)

**Table 4:** Multivariable analysis between ocular and systematic factors with average pRNFL grayscale value.

Variables, n = 397	β	*p*-value
Age (y)	− 0.053	0.002
Sex (% male)	− 0.064	0.887
Spherical equivalent (D)	–	–
Best-corrected visual acuity (logMAR)	− 5.582	0.087
Axial length (mm)	− 0.664	0.003
IOP (mmHg)	–	–
Visual field MD (dB)	–	–
pRNFL thickness (um)	0.002	0.953
RPE grayscale value	0.372	< 0.001
ImageQ	0.658	< 0.001

## Discussion

This study reported a normative pattern of pRNFL grayscale density parameters and evaluated its determinants in normal Chinese eyes measured with SD-OCT. Our data suggest the need for an age-specific normative database for pRNFL grayscale value measurement when grayscale value be used as an imaging marker to differentiate normal eyes from glaucomatous eyes in the future.

Previously published studies show that OCT grayscale value changes in several ocular diseases. Ozdemir et al. found that the grayscale value of the inner retina increases in retinal artery occlusion^12^. Some authors also suggested using OCT profiles' light reflectivity to differentiate degenerative from the exudative macular disease^13^. So far, the commercial OCT devices did not provide software to measure the optical intensity (or original signal) directly. Chen et al. proposed a method to interpret OCT data as grayscale images^14^. They found the retinal layers' optical intensities were affected by the image quality. Our previous study showed that optical intensity (measured as grayscale value) in macular was affected by image quality and age in normal subjects using a similar image analysis method^15^. The pRNFL consists of axons of all retinal ganglion cells, which were early affected by glaucomatous damage. pRNFL parameter measurement is, therefore, the most commonly utilized OCT parameter for clinical glaucoma assessments^16^. Several studies showed that pRNFL thickness parameters outperformed macular thickness parameters for the diagnosis of glaucoma^17, 18^. In our previous study, we reported ﻿pRNFL thickness in four different sectors with the area under the curve (AUC) between 0.661 and 0.938. Similar results have also been reported by other authors using different SD-OCT modalities^19, 20^. As OCT using light reflected from the RNFL to assess the thickness change, knowledge of such RNFL grayscale value can improve our understanding and detection of glaucomatous damage or progression. Huang et al. demonstrated pRNFL reflectance value decreases before thickness changes in RNFL using a glaucoma rat model^21^. Vermeer et al. further reported the highly significant difference of pRNFL attenuation coefficient between normal and glaucomatous eyes^4^. All these studies suggest optical value as a new clinical tool for diagnosing and monitoring glaucoma. Although there are differences in pRNFL grayscale value measurement compared with the previous studies, the present study extends this body of work by providing normative SD-OCT data on pRNFL grayscale value profile in nonglaucomatous eyes with a larger sample size.

In this study, the pRNFL grayscale value decreased with increasing age. Using Stratus (Carl Zeiss, Oberkochen, Germany) SD-OCT, Chen et al. reported the average RNFL thickness decreased by 4.97 μm per decade in the Chinese population^22^. Our previous study also demonstrated RNFL ﻿grayscale value in the macular area was independently affected by age. Based on histological studies, Repka et al. showed the effect of age on mean optic nerve fiber axonal diameter in normal eyes^23^. Several other studies also estimated the age-related loss of retinal ganglion cells (RGCs) (7209 cells per year)^24, 25^. As RNFL is often used as a surrogate for RGCs' content, the pRNFL grayscale value decrease may be due in part to axonal shrinkage, selective loss of large nerve fibers, or redistribution of fiber diameter. In a population-based study, Wagner et al. reported the pRNFL profiles are related to individual ocular and systemic parameters^26^. We, therefore, included axial length into multivariable analysis and revealed a statistically significant association between lower average pRNFL grayscale value and longer axial length. An increased axial length leads to a temporal shift pRNFL and thinner pRNFL thickness^27^. We did involve high myopic subjects in the current study. Further studies are needed to investigate correlations between the pRNFL grayscale value and individual ocular and systemic parameters, like refractive errors.

﻿It is interesting to note that pRNFL thickness is not an independent determinant of pRNFL grayscale value after adjusting other cofactors, like age, RPE grayscale value, and image quality. As the grayscale value has the property of less spatial variation, some authors suggested this parameter may prove to outperform or at least complement RNFL thickness measurements for diagnosis and monitoring glaucoma. Our results also imply the grayscale value may be used as an additional OCT parameter for glaucomatous structure–function research in the future. It is still controversial how to correct spatial fluctuations of the incident light intensity and ocular opacities during quantitative measurement of grayscale value. Vermeer et al. suggested using the RPE as the reference layer to normalize measurement. Our study also found RPE grayscale value is an independent factor with pRNFL grayscale value in multivariable analysis.

Our results also showed that the pRNFL grayscale value was the lowest in nasal sectors. In previous studies, using different image processing techniques, the optical characteristics (optical intensity, attenuation coefficient, or birefringence) also showed angular dependency in healthy subjects^28–30^. Because of the cylindrical nature and parallel arrangement of RNFL, grayscale value is expected to depend highly on the laser's incident angle. It is possible that pRNFL decreases rapidly as the nerve fiber descends more perpendicularly into the optic nerve at the nasal sector.

This cross-sectional study has some limitations. First, our study was a hospital-based but not a population-based study. Therefore, our results might not represent the general population. Further studies using a large and more ethnically diverse population are warranted. Second, pRNFL parameters were measured with Topcon SD-OCT in the current study. It had been reported that, in both normal and glaucomatous eyes, there have significant differences in pRNFL parameters' value obtained by different SD-OCT modalities^31^. The investigation, including grayscale value measured from other SD-OCT modalities, is needed to build up a normative database. Finally, in the current study, the image analysis was not performed on the actual linear reflectivity signal extracted from the device. The measurement may be further affected by image analysis, like preprocessing customized software or adjusting to the detector gains. Using the same image analysis, we demonstrated the diagnostic capability of pRNFL thickness to detect glaucoma in our previous study. Compared to the above-mentioned study, the pRNFL grayscale value can also achieve good diagnostic performance accuracy of 0.87 (95% CI 0.84–0.90) (Supplementary Tables S1 and S2).

In conclusion, we provided normative SD-OCT data on pRNFL grayscale value profile in nonglaucomatous eyes. Our data demonstrated that lower pRNFL grayscale value was independently correlated to older age, lower RPE grayscale value, and lower ImageQ. These determinants should be considered in interpreting this imaging marker when deployed in glaucoma assessment in the future.

## Supplementary information

Supplementary information.
