# Supplementary material for: Determinants of peripapillary retinal nerve fiber layer’s grayscale value in normal eyes by spectral domain optical coherence tomography
Source: Sci Rep. 2021 May 5;11:9577. doi: 10.1038/s41598-021-88604-x (PMC8100177; doi:10.1038/s41598-021-88604-x)
Supplement: Supplementary file 1 — Supplementary information. [file 41598_2021_88604_MOESM1_ESM.docx]

**Determinants of Peripapillary Retinal Nerve Fiber Layer Optical Intensity in Normal Eyes by Spectral Domain Optical Coherence Tomography**

Xiaolin Xie^1^ MSc, Binyao Chen^1^ MSc, Jianling Yang^1^ MSc, Chukai Huang^1^ Ph.D., Kunliang Qiu^1^ Ph.D., Ce Zheng^2*^ M.Med, PhD., Mingzhi Zhang^1*^ MSc

1: Joint Shantou International Eye Center of Shantou University and the Chinese University of Hong Kong, Shantou University Medical College, Shantou, Guangdong, China

2: Department of Ophthalmology, Xinhua Hospital, ﻿Affiliated to Shanghai Jiaotong University School of Medicine, Shanghai, China

Emails: Ce Zheng (zhengce@xinhuamed.com.cn); Xiaolin Xie (xiexl@jsiec.org); Binyao Chen (cby@jsiec.org), Jianling Yang (yjl@jsiec.org), Chukai Huang (hck@jsiec.org), Kunliang Qiu (qkl@jsiec.org); Mingzhi Zhang (zmz0754@126.com)

Corresponding Author: Ce Zheng and Mingzhi Zhang contributed equally to this works.

Dr. Ce Zheng (zhengce@xinhuamed.com.cn)

Department of Ophthalmology, Xinhua Hospital, Affiliated to Shanghai Jiaotong University School of Medicine, Shanghai, China

Telephone: +86 15000569288; Fax: +86 2052976298

Dr. Mingzhi Zhang (zmz0754@126.com)

Joint Shantou International Eye Center of Shantou University and the Chinese University of Hong Kong, Shantou University Medical College, Shantou 515000, China.

**Financial Disclosures:** None

**Keywords**: Spectral Domain Optical Coherence Tomography, Peripapillary Retinal Nerve Fiber Layer, Optical Intensity

**Word Count:** 2439

Table 1: ﻿Comparison of pRNFL parameters between normal and glaucoma subjects

|  | Normal (n=394) | Glaucoma (n=153) | | *p** |
| --- | --- | --- | --- | --- |
| Parameters | Mean ± SD | Mean ± SD |  | |
| pRNFL thickness | 106.68 ± 8.89 | 78.10 ± 20.39 | ＜0.001 | |
| pRNFL optic intensity | 164.82 ± 5.69 | 134.18 ± 20.03 | ＜0.001 | |

*p**: comparison between glaucomatous and normative eyes

Table 2: The diagnostic performance of mean pRNFL optic intensity as compared with mean pRNFL thickness to detect glaucoma.

|  | Accuracy (95% CI) | Sensitivity (95% CI) | Specificity (95% CI) |
| --- | --- | --- | --- |
| Mean pRNFL thickness | 0.89 (0.86 to 0.92) | 0.84 (0.81 to 0.87) | 0.92 (0.89 to 0.94) |
| Mean pRNFL optic intensity | 0.87 (0.84 to 0.90) | 0.82 (0.79 to 0.85) | 0.90 (0.88 to 0.93) |
